# Bacteriophages of *Myxococcus xanthus*, a Social Bacterium

**DOI:** 10.3390/v10070374

**Published:** 2018-07-18

**Authors:** Marie Vasse, Sébastien Wielgoss

**Affiliations:** Institute of Integrative Biology, ETH Zürich, Universitätstrasse 16, 8092 Zürich, Switzerland

**Keywords:** myxophage, myxobacteria, social evolution

## Abstract

Bacteriophages have been used as molecular tools in fundamental biology investigations for decades. Beyond this, however, they play a crucial role in the eco-evolutionary dynamics of bacterial communities through their demographic impact and the source of genetic information they represent. The increasing interest in describing ecological and evolutionary aspects of bacteria–phage interactions has led to major insights into their fundamental characteristics, including arms race dynamics and acquired bacterial immunity. Here, we review knowledge on the phages of the myxobacteria with a major focus on phages infecting *Myxococcus xanthus*, a bacterial model system widely used to study developmental biology and social evolution. In particular, we focus upon the isolation of myxophages from natural sources and describe the morphology and life cycle parameters, as well as the molecular genetics and genomics of the major groups of myxophages. Finally, we propose several interesting research directions which focus on the interplay between myxobacterial host sociality and bacteria–phage interactions.

## 1. Introduction

The massive *de novo* generation of viral genome and metagenome data from environmental and clinical sources [1,2] as well as the surging interest in molecular and medical applications derived from research on phage therapy [3] and bacterial CRISPR-Cas (Clustered Regularly Interspaced Short Palindromic Repeats and CRISPR associated system) immunity in eubacteria and archaea [4] have reinvigorated scientific interest in phage ecology and evolution [5]. As we begin to appreciate the astounding diversity of viromes, we divert our focus from the few described phages used as laboratory workhorses [6] to the mind-boggling estimated number of 10^31^ non-model phage particles in nature [7]. This enormous diversity offers great potential for the emergence of novel applications and concepts [2], as depicted by the elucidation of phage–phage communication in viruses of *Bacillus* spp. [8], or the finding of a new class of lysogeny switches in mycobacteriophages [9].

**Figure 1:**
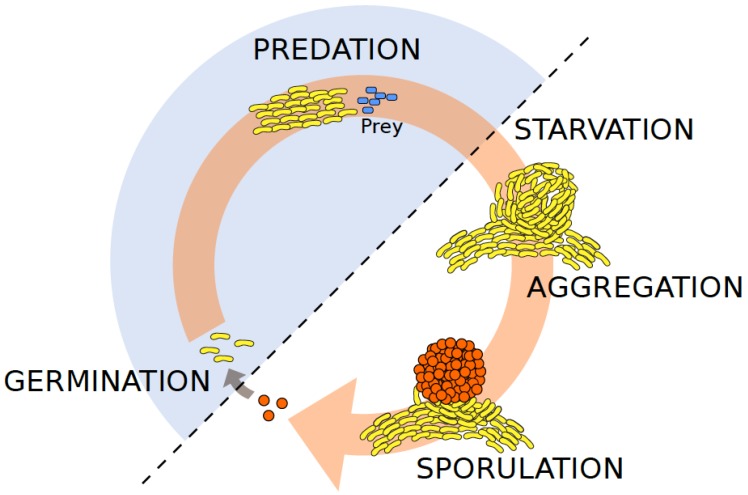
Schematic life cycle of the social myxobacterium *Myxoccocus xanthus*. The life cycle of *M. xanthus* (yellow-pigmented rods) is governed by the availability of food sources such as organic compounds or bacterial prey cells (blue rods). Under ambient nutrient-rich conditions, *M. xanthus* cells engage in group-level predation for feeding and rely on different (both social and solitary) motility systems. It is in this vegetative stage (highlighted as shaded blue half-circle), that cells are most susceptible to phage adsorption, such as shown for Mx1 [34]. In contrast, under nutrient limitations, *M. xanthus* kin groups undergo multicellular development that typically involves fruiting body formation in which a fraction of cells forms stress-resistant spores (orange spheres). Phages are unable to infect their hosts in the spore stage, but may infect cells after spores have germinated [34].

In this review, we will broadly summarize what is known about the bacteriophages of the myxobacteria, which are important model organisms for understanding the evolution of multicellularity and cooperation. Myxobacteria are a monophyletic clade within the delta-proteobacteria, and are highly diversified [10,11] and globally distributed in both terrestrial soils [12] and marine sediments [13,14]. Their life cycle is governed by the availability of food sources (Figure 1) and they either feed on organic compounds (such as cellulose) or microbial prey cells by extracellular lysis [15]. However, some members have also evolved parasitic lifestyles, e.g., in salt water algae [16]. As such, they represent important constituents of microbial ecosystems [17]. Key features of group living, including motility [18,19], and multicellular development [20] (Figure 1) have been elucidated in great mechanistic detail for *Myxococcus xanthus* which “has become the *Escherichia coli* of Developmental Biology” [21]. Recently, *M. xanthus* has also emerged as a model for the study of the fundamental parameters governing the evolution of multicellularity and cooperation in bacteria [22,23,24,25,26]. Cooperative swarming and multicellular development are also variable in *M. xanthus* isolates from nature [27,28,29], and this species is one of the few bacteria whose phylogeographic patterns have been elucidated at precisely defined metric scales using both Multi Locus Sequence Typing (MLST) [30,31,32] and whole genome data [33].

In contrast to their bacterial hosts, very little is known about the myxophages. As is true for most bacterial systems, phages of the myxobacteria have been mainly used as molecular tools for strain construction to study host biology [35]. For instance, the generalized transducing myxophages enabled strain construction for the study of the genetic mechanisms underlying traits such as gliding motility (using Mx8 [36]), and multicellular development (using both Mx4 and Mx8 [37]) to name just a few examples. Several excellent reviews on utilizing myxophages and their genes as genetic tools for transduction and transformation already exist (e.g., [35,38]), and we will thus only briefly mention this aspect throughout our text.

In our review, we first highlight the difficulties encountered in isolating and consistently naming myxophages, and propose a general naming scheme for both existing and novel myxophages. We then describe the main groups of myxophages, phage remnants, and prophages with regard to morphology, biology, and molecular genetics and genomics. Finally, we conclude this review by outlining several interesting research questions related to the interplay between myxobacterial host sociality and bacteria–phage interactions.

## 2. Isolation Procedures of Myxophages from Nature

To study the genetic bases of phenotypic traits in myxobacteria, including their characteristic multicellular behaviors (Figure 1), several research groups have devoted their efforts to isolating general transducing phages with myxobacterial host strains used in the laboratory. In 1966, Burchard and Dworkin successfully isolated and described the first *M. xanthus* myxophage (virulent phage “MX-1”) from cow dung, but failed to yield bacteriophages from soil, sewage, or water using classical isolation techniques [34]. It was not until around a decade later that general transducing phage types could be successfully isolated. In particular, phage Mx4 was isolated from farmyard manure, and amplified on the indicator strain *M. xanthus* DZ1 [39], while most other phages (Mx41, Mx43, Mx8, Mx81, Mx82, Mx9) derived from the supernatants of liquid cultures of fruiting strains were originally isolated from cultivated soil ([39]; using Singh’s method [40]). To check for the presence of phages, purified supernatants were plated on soft agar with indicator strains (Table 1; [39]). In summary, techniques for phage isolation include one or more cycles of bacterial growth in liquid broth or on agar surfaces with subsequent (1) killing of bacteria with chloroform thereby releasing intracellular phages [39,41], (2) filtration and centrifugation [42], and/or (3) centrifugation in sucrose gradients [41,43]. As typical for many phages, bivalent Ca^2+^-ions were reported to increase resulting phage titers [34], and mitomycin C was used to trigger the lytic phase of temperate phages and facilitate their excision [42]. Finally, plaques of several phages (Mx1*l*3 and Mx8*l*1) were discovered on plates of mutagen-treated cell cultures containing Mx4*l*6 [42]. The authors hypothesized that such plaquing phages resulted from recombination events between several defective or active prophages present in the host strain. They further suggested that superinfection by damaged phages could give rise to new phages [42]. Many of the later isolated myxophages are morphologically and serologically very similar to the well-described myxophages (see section below), even though they come from geographically distant samples [41,42]. Such sampling redundancy, along with the fact that many myxophages of groups Mx4, Mx8, and Mx9 do not plaque on the strains they were originally isolated from [39], suggests that sampling strategies and isolation protocols need careful refinement in order to isolate and characterize entirely novel myxophages. Moreover, culture-independent methods [44] such as metagenomics and whole genome sequencing of host genomes should help to reveal myxophage diversity (including prophages).

## 3. Nomenclature of Myxophages

Myxophages have primarily been named following the nomenclature laid out during the International Meeting on Myxobacteria in 1976 [39]. There, it was decided that for phages capable of infecting myxobacteria, names should be composed of a first part with two letters that signify the myxobacterial host species name (e.g., “Mx” for *M. xanthus*) followed by a single digit which corresponds to the distinct serological type of the phage. The first phage ever to be described for a distinct serotype group (called the “prototype-phage”) was then to be adopted as the general name of that group. For each subsequently isolated and characterized phage belonging to a previously characterized group, additional digits should be added, e.g., Mx4-like phages Mx41 and Mx43 were described in [39], shortly after the first-ever description of a phage of serotype 4, Mx4 [45,47]. This nomenclature, however, has been adopted rather inconsistently over the years, as can be easily depicted by the original names of the four main groups of myxophages hitherto characterized to date: MX-1, MX4, Mx8, and Mx9 (Table 1 and Table 2). Moreover, phages named prior to the above-mentioned meeting in 1976 often have names that deviate from the nomenclature. Indeed, after the isolation of Mx1 [34], a second paper [41] characterized a range of novel myxophage particles very similar to Mx1 but used an entirely different naming scheme. Here, the italicized Greek symbol “phi” (i.e., ϕ) was followed by either a single letter or a digit, each referring to the particular *Myxococcus* strain from which they were isolated (e.g., ϕm originates from *Myxococcus fulvus* M18, and ϕ2 derives from *Myxococcus virescens* V2 [41], see Table 2 for a complete list of ϕ phages). As a remark, “phi” has been converted to “f” (such as in “fm” and “f2”) in the publically available phage catalogue “Bacteriophage Names 2000” [48]. Later, Rodrigues et al. [42] reported a larger set of phage isolates, all resembling known main groups, but naming them according to a variation of the official nomenclature. They used a letter to indicate the location of the lab in which they were isolated, i.e., *l* for Leeds (UK) or *k* for Karlsruhe (D), capped off by a set of running digits. Phage Mx1*l*3, for example, is the third phage of serotype group Mx1 isolated in Leeds (see Table 2 for a complete list). Finally, several phages that have been isolated from *Myxococcus virescens* were first grouped according to their resemblance to *M. xanthus-*specific phages, but named as “Mv”-type phages, e.g., Mv-8 g1 [49] is reminiscent of myxophage Mx8. Since phages reported in [41,42,49] have not been deposited in publically available strain collections, it appears they have been lost.

In our opinion, the original nomenclature from 1976 [39] is straightforward if followed stringently, but would limit the overall number of potential myxophage groups to 10 (if one includes Mx0). Hence, we propose the following general extension of the myxophage naming scheme:

(1) A phage found to infect a certain myxobacterial species is assigned letters composed of this initial host’s genus and species, respectively; e.g., phages found to infect *M. xanthus* are assigned Mx, and those of *M. virescens* should be named Mv. In some cases, ambiguity will arise due to identical initials. In such cases more letters should be used, e.g., Cfu and Cfe for *Cystobacter fuscus* and *Cystobacter ferrugineus*, respectively. If multiple host species are known, the myxophage name may refer to the original source host (if applicable), or arbitrarily to either of the host species if directly derived from environmental samples (such as soil).
(2) The letters are immediately followed by a distinct serotype category expressed as a running number. This will both help to account for existing phage names and also allow for an unlimited number of novel categories. As before, the first phage describing a novel serotype group is called the prototype-phage and is adopted as representative of that novel serogroup.
(3) For non-prototype phages, the name is finally capped off by a running number separated by a dash from the main group in order of first description, e.g., novel phage isolates that are serologically classified as “Mx1-like” are labeled Mx1-1, Mx1-2, etc.

With this new naming convention, we also consider to appropriately rename already isolated myxophages, as summarized in Table 3. For practical reasons, we propose that all phages keep their original numbers, and new phages are assigned numbers starting from the last labeled phage. Lost phages can be integrated formally at a later stage in case they are retrieved (i.e., myxophages cited in [41,42] including the ϕ-myxophages and Mx1*l*3, etc.).

## 4. Characterization of the Main Groups of Myxophages

**Figure 2:**
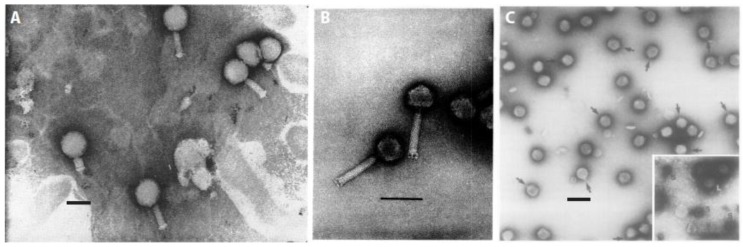
Micrographs of distinct serological types of myxophages. (**A**) Phage Mx1 (Reproduced with permission from [34]); (**B**) phage Mx4 (Reproduced with permission from [47]); and, (**C**) phage Mx8 (Reproduced with permission from [39]). Inset, large and small phage particles after centrifugation are shown. Phage Mx9 is described as morphologically very similar to Mx8 [39]. Scale bars represent 100 nm each.

In the following, we summarize knowledge about the main groups of myxophages (some of which are depicted in Figure 2). It is organized into seven parts: the first four subsections introduce the four distinct serogroups of myxophages isolated to date; the fifth part describes prophage *Mx alpha*; the sixth subsection highlights previously misclassified myxophages; and the seventh part lists phage remnants and bacteriocins encoded in the genomes of myxobacteria. Finally, we would like to highlight that we published an editable database for sharing and exchanging relevant information and resources within the bacteriophage community [53].

### 4.1. The Mx1-Like Group

The Mx1-like group (Table 1 and Table 2) contains a total of seven phage isolates, comprising Mx1, the first isolated phage capable of infecting *M. xanthus* [34], as well as Mx1*l*3 [42] and a set of five of the aforementioned ϕ phages (ϕa, ϕb, ϕm, ϕv, ϕ2) [41]. Morphologically, these phages have been classified as *Myoviridae* of the A1-group [48] with isometric polyhedral heads and long and thick contractile tails. In morphology, they are roughly similar to coliphages T2 and T4 [43]. Phage Mx1, the best-studied of these phages, consists of a particle of about 175 nm length, with a 100-nm long contractile tail and a 75-nm-wide head [34]. It has a latent period of 120–150 min at 30 °C in *M. xanthus* and a final burst size of ~100 [34]. The adsorption occurs mainly within the host-cell polar regions [50] and is improved by adding 2% Difco Casitone, 10^−3^ M CaCl_2_, and 10^−2^ M K_2_HPO_4_-KH_2_PO_4_ buffer (equilibrated to pH 7.6) [34]. Host cells become spherical following adsorption [50]. Mx1 is resistant to sonication [54]. All Mx1-like phages are virulent and capable of infecting vegetative cells of various strains of the genus *Myxococcus*. Phage Mx1 infects *M. xanthus* FB_tan_, *M. virescens* V2, and *M. fulvus* M upon which it forms clear plaques [34,41,43], but does not infect *M. fulvus* MF [34]. It was noted that Mx1, ϕa, ϕb and ϕm are morphologically very similar to one another despite their distinct sampling locations on either side of the Atlantic [41], which was interpreted as a sign for their common origin. In contrast to all other described Mx1-like phages, Mx1*l*3 is also capable of stably transducing different markers, including rifampicin resistance [42].

### 4.2. The Mx4-Like Group

Based on isolation success, the Mx4-like group (Table 1 and Table 2) is the largest group of myxophages as it contains 20 distinct members. It comprises Mx4, isolated from farmyard manure in California [45], phages Mx4-1 and Mx4-3 derived from culture supernatants [39], and a large group of 17 Mx4-like isolates labelled *l* (Leeds) and *k* (Karlsruhe) [42]. Based on morphology, Mx4-like phages were classified as *Myoviridae* of the A1-group [48]. Mx4-like phages (or mutants thereof, Table 1), are typically described as generalized transducing phages with latent infections [39,45]. Importantly, despite the fact that plaques appear turbid on indicator strains, the original Mx4 isolate is still considered a virulent phage since no lysogenic bacteria could be isolated upon infection [45,55]. The host range of the original Mx4 phage isolate is limited to specific *M. xanthus* mutants [39,45], and can be extended to other lab strains by a phage host range mutation (*hrm*-1; originally derived and contributed by Wolfner (unpublished) and described in [45]). When the latter was combined with another mutation to temperature sensitivity (*ts27*; reducing killing of transductants), a generalized transducing strain, Mx4 *ts27htf-1hrm-1* [45], of very high efficiency, was retrieved ([45]; Table 1). Mx4 has a latent period of about 180 min at 35 °C in strain *M. xanthus* DZ1 and a burst size of 75 [45]. Its icosahedral head is about 60 nm in diameter and it has a long and thin contractile tail [45,47]. Mx4-like phages have been isolated from different locations and showed various host ranges and transduction efficiencies suggesting a wide geographical repartition for this group [42].

### 4.3. The Mx8-Like Group

Phages of the Mx8-like group (Table 1 and Table 2) belong to the *Podoviridae* of subtype C1 [48] and show morphological similarity to *Salmonella* phage P22 [39,51]. They are characterized by isometric, polyhedral heads and short, non-contractile tails [39]. So far, a total of four phages have been described: Mx8, Mx8-1, Mx8-2 were derived from the supernatants of liquid cultures of fruiting *M. xanthus* strains sampled from soil [39], and Mx8*l*1 was discovered on plates of mutagenized Mx4*l*6 [42]. They are all generalized transducers of both wildtype and mutant strains of *M. xanthus*. Mx8 proved to be the most pivotal general transducing agent in the molecular genetic toolkit for myxobacterial research (e.g., summarized in [38]). Importantly, the original stock consisted of several morphological subtypes, which could be distinguished based on plaque morphology ([46]; and listed in Table 1). Moreover, based on infection experiments of different host lysogens of *M. xanthus* DZ1, it was later shown that Mx8 and Mx8-2 are in fact independent isolates of the same temperate phage [51].

### 4.4. The Mx9-Like Group

The Mx9-like group (Table 1 and Table 2) is the smallest group of myxophages and consists of only a single phage, Mx9, isolated from the supernatant of a liquid culture of a fruiting *M. xanthus* strain sampled from soil [39]. Mx9 is serologically different from other temperate phages [39], but is morphologically highly similar to Mx8-like phages and to *Salmonella* phage P22 [39]. It is a small phage belonging to the *Podoviridae* of subtype C1 [48], with a very short or absent tail. Mx9 is a general transducing phage [39], and integrates at one of two *attB* sites (*attB1* and *attB2*) in the *M. xanthus* host genome using a single phage-encoded gene, *int* [52].

### 4.5. Prophage Mx Alpha

An endogenous myxophage, *Mx alpha*, has been detected in *M. xanthus* strain YS [56,57], a single clonal isolate from parental strain FB, the ancestor of most standard laboratory strains in use [58]. Coincidentally, *Mx alpha* has been found to be the causative agent of transmitting Tn5 transposon tags between different *M. xanthus* strains [56]. Such genetic exchange was mediated via specialized transduction with tail-less phage particles of ~35 nm in diameter [56]. Physical mapping indicated that a single *Mx alpha* prophage unit approximately covers about ~80 kilobase pairs (kbp) in the *M. xanthus* host genome, and that it is widespread among many different isolates, sometimes in multiple copies [57,58]. Since the *Mx alpha* phage heads are too small to carry the entire prophage’s genetic information of ~80 kb, it is assumed that entire units are transduced by multiple infections [57]. There exist three successive *Mx alpha* copies in both *M. xanthus* FB and YS in a confined genomic region of ~300 kbp. However, several laboratory strains which together with YS are all derived from the same parent, FB [58], lack at least part of this region. In particular, strain DK1622, which is a widely used wild-type laboratory strain of *M. xanthus*, only harbors a single unit, lacking around 222 kbp of that region [57,58,59]. Another commonly used laboratory strain, DZ1, was found to lack the *Mx alpha* diagnostic marker [58], which hints at a deletion. Not surprisingly, the genomic region harboring the *Mx alpha* prophages (located between 2.1–2.25 megabase pairs (Mbp) of the *M. xanthus* DK1622 reference genome; [60]) is among the most highly variable genomic regions in natural isolates of *M. xanthus* [33]. Crucially, the latter study found that the presence or absence patterns of different genes in that region (several of which are phage-related) are highly correlated with kin discrimination behavior among these isolates [33]. This finding is in line with a parallel finding that linked the presence or absence of whole copies of *Mx alpha* to antagonistic social behavior among closely related *Myxococcus* laboratory strains [58]. Among the genes in this region, *MXAN_1899* was singled out as a particularly interesting candidate gene based on short sequence homology to the CdiA C-terminal toxin domain [33]. Recently, this gene, now referred to as *sitA3*, has been shown to represent a toxin-immunity system involved in *traA*-dependent killing behavior among *Myxococcus* strains [61]. Moreover, while DK1622 only carries a single toxin in its sole remaining *Mx alpha* unit, its parent, DK101, contains two additional *Mx alpha* units carrying *sitA1* and *sitA2* toxin-immunity systems, respectively [61].

### 4.6. Misclassified Phages

At least two additional phages were initially described as bacteriophages of myxobacteria in the literature [62,63]. They infect the bacterial pathogen *Flavobacterium columnarae*, the causative agent of columnaris disease in freshwater fish [64]. Importantly, while the latter bacterium was formerly classified as a myxobacterium named *Chondrococcus columnaris* based on morphological criteria (summarized in [65]), it is now classified in a taxonomic group distinct from the myxobacteria [64]. We therefore conclude that phages capable of infecting *Chondrococcus columnaris* are in fact not myxophages (even though they are labeled as such), i.e., “Myxophage” [62] and “Myxophage C2” [63].

### 4.7. Phage Remnants and Bacteriocins

In addition to the five ϕ phages, Brown and colleagues also described a range of phage-like particles in the culture supernatants of several *Myxococcus* species that were phenotypically similar to myxophage Mx1 [41]. These particles, however, could not be propagated in bacterial hosts and electron micrographs indicated them to be morphologically largely defective [41]. The authors further observed that several bacterial species that grew together with microcolonies of myxobacterium *M. virescens* V2 were either completely lysed (in the case of *Pseudomonas fluorescens* 4b) or showed signs of cloudy plaque formation (in the case of *Cytophaga johnsonae* and *Salmonella typhimurium* ST908; [41]). The authors speculated that this lytic factor could be related to xanthacin, a bacteriocin found in *M. xanthus*, that is highly resistant to enzymatic degradation and heat [66]. Preparations of xanthacin show vesicular structures of ~50 nm in diameter [66]. Unlike the lytic factor found by Brown and colleagues [41], xanthacin is not active against the producing strain [66]. Structures resembling either phage tails or sheaths have also been found to be produced by myxobacterium *Archangium violaceum*, and production of these structures could be enhanced following treatment with mitomycin C [67].

## 5. Genomic Features of the Main Myxophage Groups

**Figure 3:**
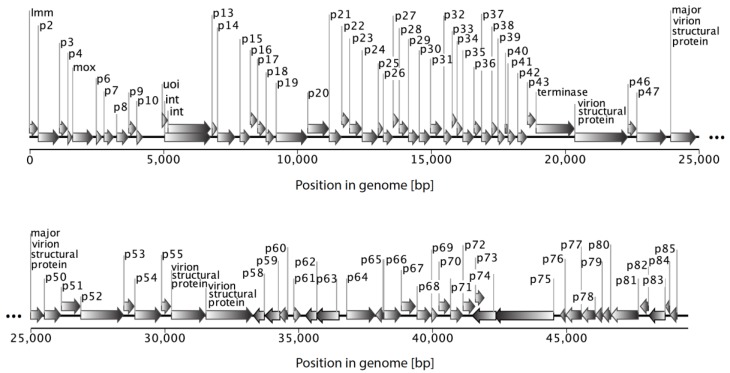
Organization of the myxophage Mx8 genome (size ~49.5 kbp; Genbank ACC # AF396866). Each of the 85 predicted protein-coding genes are depicted by arrows and labelled with connectors.

All known myxophages are linear double-stranded DNA viruses (Table 2). While the majority of these phages have a similar GC-content compared to their *Myxoccocus* hosts (i.e., ~68% for *M. xanthus* [60]), virulent myxophage Mx1 deviates from this pattern: its genome has a GC-content of close to 50% [34,68], and potentially contains several different minor pyrimidine components aside from C or T, which were never unequivocally identified [34,69]. The genome sizes vary widely among the different myxophages and were mostly measured in form of molecular weight (MW) estimates [34,39,42,46,47,51,68]. In the following, we summarize and convert MW into modern units, i.e., kilobase pairs (kbp). The genome size of Mx1 has been estimated only based on buoyant density centrifugation methods and ranges between 130–149 × 10^6^ Daltons (g/mol). The lower estimate translates to a genome size of 163 kbp, when using T-even coliphage T2 as a measure [70]. The genome sizes of Mx4-like phages have also been reported in MW units ranging from 34.8 to 42.3 × 10^6^ Daltons [42,47]. It is however possible to convert these data based on the genome size of phage Mx8. The latter has a fully sequenced genome of 49.5 kbp (Figure 3; Genbank ACC # AF396866; [51,71]). This translates into 37.1 × 10^6^ Da, and given that Mx4 and Mx8 have comparable GC contents of ~68% [39,47], it follows that the genomes of Mx4-like phages should range between 46 and 56 kbp (see [42]).

Molecularly, the genome of Mx8 is the best understood amongst myxophages. It is widely used for strain construction using molecular tools thereof for transduction or transformation [35,38]. Mx8 integrates as a stable prophage through site-specific recombination between *attP* in the phage genome [71], and either of two neighboring Mx8 phage attachment sites, *attB1* and *attB2*, situated in the host chromosome [72]. The attachment site *attP* is situated within the coding sequence of the integrase gene *int* (Figure 3; [72,73]) and the integration of the Mx8 prophage thus creates a recombinant gene resulting in a new 3′ end of *int*. Magrini and collaborators [74] showed that this event did not inactivate the integrase but modified its specificity in such a way that the integrase protein is able to recombine with several phage attachment sites, with varying efficiencies. A similar principle of integration via recombination between specific attachment sites in phage and host genomes has been elucidated for phage Mx9 [52]. This constitutes a molecular switch to convert a lysogen from the lysogenic to the lytic phase and enables a tightly controlled coordination of the rates at which prophages are excised from the host’s genome. The molecular elucidation of integration via recombination has led to the construction of widely utilized plasmids [73] that carry a selectable genetic marker such as kanamycin resistance, along with a locus of interest, e.g., used in complementation studies [35,38].

Beside the integrase gene *int*, the function of genes encoding: (1) superinfection immunity, *imm* [51,74]; (2) helix-turn-helix (HTH) protein, *uoi* [49]; and (3) DNA adenine methylase, *mox* [75], were previously described (see Figure 3 for the location of genes in the Mx8 genome).

## 6. Concluding Remarks and Future Research Directions

Since the late 1960s, more than 35 myxophages have been isolated, characterized, and classified into four main serological groups. Among these, several generalized transducing phages of *M. xanthus* strains have been derived, of which Mx4 and Mx8 are the most widely used molecular tools [38]. Moreover, Mx8 is the only completely sequenced phage to date (Figure 3). Most myxophage studies are relatively old (about 30–50 years) and therefore modern genetic and genomic data are scarce. Here, we compiled the present knowledge on the myxophages in the hope that this review will fuel further research on their biology, genetics and genomics. In particular, a comparative analysis of the different myxophage genomes would improve our mechanistic understanding of their genomic architecture, genetic exchange mechanisms, genetic bases for host resistance, and phage infectivity. Finally, parallel sampling of myxobacteria and their viral parasites in strictly defined biogeographic contexts should help shed a new light on the coevolutionary trajectories of phage-bacteria interactions in nature.

*M. xanthus* is a model bacterium for the study of developmental biology and social evolution because most stages of its life cycle rely on cooperation (Figure 1). The isolation of myxophages therefore offers an interesting opportunity to study the interplay between bacterial sociality, multicellular development and bacteria–phage interactions both at ecological and evolutionary time-scales. In the following, we outline promising research perspectives. First, *M. xanthus* displays two distinct forms of motility: motor-driven “adventurous” gliding of single cells [19,76] and “social” twitching of cell groups powered by a type IVa pilus extension and retraction machine [18]. While motility, in some cases, has been shown to be associated with higher resistance to phages [77,78], the effect of phage selective pressure on the evolution of different motility types remains unknown. Second, *M. xanthus* and myxophages could enable investigations on the eco-evolutionary dynamics of microbial predation, food webs and multi-interaction networks. *M. xanthus* cell groups collectively prey on a wide range of bacteria. Thereby, prey-derived nutrients are released in the communal foraging environment, which therefore function as public goods available to all members of the predatory group [15]. While it is currently unknown how bacteria–phage interactions could affect predatory behaviors in *M. xanthus*, exciting questions have been raised with respect to the evolution of multi-level predator–prey interactions, in which *M. xanthus* is both a predator of bacterial prey and a prey of myxophages. For example, in the latter scenario, the secretion of exopolysaccharides may both mediate predator motility but impede phage attachment, such that more effective predation could be positively correlated with higher phage resistance. Third, and in relation to the former perspectivepoint, only little is known about the molecular mechanisms that confer phage resistance in myxobacteria. For example, several mutations in genes encoding for the components of the lipopolysaccharide (LPS) decrease phage sensitivity in *M. xanthus* [79]. Importantly, however, several other candidate loci exist in myxobacteria that are also of great relevance. For example, natural isolates of *M. xanthus* encode multiple CRISPR-Cas-systems [33]. These loci are typically involved in bacterial immunity that help fend off invasive genetic elements, such as phages [80]. In *M. xanthus*, two such CRISPR-Cas systems are involved in multicellular development [81,82,83], which offers an intriguing potential link between phage immunity and social behaviors in the myxobacteria. Fourth, it would be interesting to explore how virulent and temperate myxophages affect the developmental cycle of *M. xanthus*. Indeed, Mx1 phages have been shown to decrease germination success as they infect cells at the onset of spore formation. Here, the phage DNA is trapped in the spores and a fraction of infected spores releases phage progeny [54]. Whether phages can trigger fruiting body formation or delay germination however remains to be investigated. Moreover, it is unclear how temperate phages stably remain integrated in the *M. xanthus* genome for several cycles of host sporulation and germination despite the modification of gene expression between the different phases of the bacterial developmental cycle [46]. More generally, understanding the role phages play in shaping microbial communities is paramount for understanding the function of these complex environments.

## Figures and Tables

**Table 1:** Overview of well-described bacteriophages of myxobacteria.

Original Name (New Name) ^1^	Source	Life Cycle	Transduction Capacity	Host Range among Lab Strains ^2^
MX-1 (Mx1) [34]	cow dung [34]	virulent [34]	-	*Myxococcus xanthus* strains: FB_tan_ (Fru^+^) [34], MC (Fru^−^) [34], DZ1 (Fru^−^, Mot^−^) [39], *Myxococcus fulvus* M [43], *Myxococcus virescens* V2 [43]
MX4 (Mx4) [45]	farmyard manure [45]	virulent [45]	-	*M. xanthus* strains: DZ1 (Fru^−^, Mot^−^) [45], DZF6-like (Fru^+^, Mot^+^, *Spec^r^*) [45], DK510 (Fru^−^, Mot^+^) [39]
MX4 *hrm-1*, MX4* [45]	host range mutant of MX4 [45]	-	generalized [45]	*M. xanthus* FB (Fru^+^, Mot^+^) [45]
MX4 *ts27htf-1hrm-1* [45]	generalized transduction mutant of MX4 [45]	temperate at 35 °C [45]	generalized [45]	*M. xanthus* FB (Fru^+^, Mot^+^) [45]
Mx8 (Mx8) [39]	bacterial isolates from soil sample [39]	temperate [39]	generalized [39]	*M. xanthus* strains: DZ1 (Fru^−^, Mot^−^) [39], DK510 (Fru^−^, Mot^+^) [39], DK1050 (=FB) (Fru^+^, Mot^+^) [39]
Mx8 *cd* [46]	cloudy subtype of MX8 [46]	temperate [46]	generalized [46]	*M. xanthus* strains [46]
Mx8 *cr* [46]	turbid subtype of Mx8 [46]	temperate [46]	generalized [46]	*M. xanthus* strains [46]
Mx8 *a* [46]	very turbid subtype of Mx8 [46]	temperate [46]	generalized [46]	*M. xanthus* strains [46]
Mx8 *c (clp2)* [46]	clear plaquing mutant of Mx8 *a* [46]	temperate [46]	generalized [46]	*M. xanthus* strains [46]
Mx9 (Mx9) [39]	bacterial isolates from soil sample [39]	temperate [39]	generalized [39]	*M. xanthus* strains: DZ1 (Fru^−^, Mot^−^) [39], DK510 (Fru^−^, Mot^+^) [39], DK1050 (=FB) (Fru^+^, Mot^+^) [39]

^1^ MX (=Mx), myxophage name identifier (in reference to described host *Myxococcus xanthus*); *hrm*, host range mutant; *ts*, temperature sensitive; *htf*, high transduction frequency; *cd*, cloudy plaque morphology; *cr*, plaque morphology slightly clearer than *cd*; *a*, very turbid plaque morphology; *c* (=*clp*), clear plaque morphology; ^2^ Fru^+^: fruiting strain; Fru^−^: non-fruiting strain; Mot^+^: motile strain; Mot^−^: non-motile strain; *Spec^r^*: spectinomycin resistant strain (500 µg per mL).

**Table 2:** Morphological features of well-described bacteriophages of myxobacteria.

Original Name (New Name) ^1^	Genome ^2^	Tail Length [nm]	Tail Structure	Head Diameter [nm]	Head Shape	Plaque Morphology	Similar Morphologies to Enterophages	Morphologically-Related Myxophage Isolates
MX-1 (Mx1) [34]	dsDNA [34]	100 [34]	long, contractile [34]	75–90 [34]	isometric, polyhedral [34]	clear [34]	T-even [50]	From [42]: Mx1*l*3
								From [41]: ϕa, ϕb, ϕm, ϕv, ϕ2 (ϕ = phi)
								From [49]: Mv-1 g1, Mv-1 g2
MX4 (Mx4) [45]	dsDNA [47]	118 [47]	long, contractile [47]	67 [47]	isometric, icosahedral [47]	clear [45]	T4, **λ** [45,47]	From [39]: Mx41, Mx43
								From [42]: Mx4*l*2, Mx4*l*4, Mx4*l*6, Mx4*l*7, Mx4*l*8, Mx4l10, Mx4*l*11, Mx4*l*12, Mx4*l*13, Mx4*l*14, Mx4*k*1, Mx4*k*2, Mx4*k*3, Mx4*k*4, Mx4*k*6, Mx4*k*10, Mx4*k*13
Mx8 (Mx8) [39]	dsDNA [39]	10 [39]	short, non-contractile [39]	60 [39]	isometric, polyhedral [39]	turbid [39]	P22 [39,51]	From [39]: Mx81, Mx82
								From [42]: Mx8*l*1
								From [49]: Mv-8 g1, Mv-8 g2
Mx9 (Mx9) [39]	dsDNA [39,52]	-	very short, non-contractile [39]	60 [39]	isometric, polyhedral [39]	turbid [39]	P22 [39,51]	-

^1^ MX (=Mx), myxophage name identifier (in reference to described host *Myxococcus xanthus*); ^2^ dsDNA, double stranded DNA.

**Table 3:** Applying proposed nomenclature to previously isolated myxophages.

Host Species	Original Phage Name	New Name	Phage Group
*Myxococcus xanthus*	MX-1	Mx1	Mx1-like
	MX4	Mx4	Mx4-like
	Mx41	Mx4-1	Mx4-like
	Mx43	Mx4-3	Mx4-like
	Mx8	Mx8	Mx8-like
	Mx81	Mx8-1	Mx8-like
	Mx82	Mx8-2	Mx8-like
	Mx9	Mx9	Mx9-like
*Myxococcus virescens*	Mv-1 g1	Mv1	Mv1-like
	Mv-1 g2	Mv1-1	Mv1-like
	Mv-8 g1	Mv8	Mv8-like
	Mv-8 g2	Mv8-1	Mv8-like
